# The Selenium Yeast vs Selenium Methionine on Cell Viability, Selenoprotein Profile and Redox Status *via* JNK/ P38 Pathway in Porcine Mammary Epithelial Cells

**DOI:** 10.3389/fvets.2022.850935

**Published:** 2022-04-01

**Authors:** Caichi Wu, Chang Cui, Xiaoyu Zheng, Jun Wang, Ziwei Ma, Pengwei Zhu, Gang Lin, Shihai Zhang, Wutai Guan, Fang Chen

**Affiliations:** ^1^Guangdong Provincial Key Laboratory of Animal Nutrition Control, College of Animal Science, South China Agricultural University, Guangzhou, China; ^2^College of Animal Science and National Engineering Research Center for Breeding Swine Industry, South China Agricultural University, Guangzhou, China; ^3^Guangdong Laboratory for Lingnan Modern Agriculture, South China Agricultural University, Guangzhou, China; ^4^Key Laboratory of Agrifood Safety and Quality, Institute of Quality Standards and Testing Technology for Agricultural Products, Chinese Academy of Agricultural Sciences, Ministry of Agriculture and Rural Affairs, Beijing, China

**Keywords:** antioxidant, cell viability, porcine mammary epithelial cells, organic selenium, selenoprotein

## Abstract

Comprehensive studies have been conducted to compare the effect of organic and inorganic selenium previously, but there is still limited knowledge about the difference between organic selenium (Se) from varied sources despite the widely use of organic Se in both animal and human being nutrient additives. In the present study, we systemically compared the effect of two different types of organic Se including selenium yeast (SeY) and selenium methionine (Sel-Met) on cell viability, selenoprotein transcriptome, and antioxidant status in porcine mammary epithelial cells (PMECs) and the results indicated that appropriate addition of SeY and Sel-Met both significantly promoted cell viability and up-regulated the mRNA expression of most selenopreoteins including DIOs, GPXs, and TrxRs family et al. (*P* < 0.05). Besides, two different sources of Se supplementation both greatly improved redox status with higher levels of T-AOC, SOD, and CAT (*P* < 0.05), while less content of MDA (*P* < 0.05), and reduced protein expression of cleaved-caspase-3 (*P* < 0.05) to mitigate cell apoptosis. Furthermore, the key proteins related to p38/JNK pathway including p38, p-p38, JNK, and p-JNK were apparently reduced in the groups with both of SeY and Sel-Met (*P* < 0.05). Interestingly we found that the changes induced by SeY supplementation in cell viability, selenoprotein transcriptome, antioxidative capacity, and anti-apoptosis were comprehensively greater compared with same levels addition of Sel-Met in PEMCs (*P* < 0.05). In conclusion, both SeY and Sel-Met promoted cell viability and attenuated cell apoptosis by regulating the selenoprotein expression and antioxidative capacity *via* p38/JNK signaling pathway in PMEC, but SeY has more efficient benefits than that of Sel-Met.

## Introduction

Lactating sows showed higher nutrition and energy demands due to the extensive metabolism in mammary glands to produce milk, associated with adaption of a whole animal body system. It has been reported that 632 genes related to amino acids, fatty acids, and glucose metabolism were differentially expressed in livers during lactation (1). Increased metabolic burdens in lactating sows result in great oxygen consumption, oxygen free radicals, and lipid peroxides, leading to aggravation of oxidative stress (2, 3). Comprehensive studies have reported the damage of mammary tissues caused by oxidative stress (4), and acute redox imbalance induced by failed adaption of mammary glands during the transition from pregnancy to lactation was a main cause of mastitis accompanying impaired mammary functions (5). Further studies suggested that the damages of mammary glands induced by severe oxidative stress negatively impacted offspring's systemic redox balance and health, as well as boosted inflammatory response through breast feeding (6). Therefore, it is of importance to relieve oxidative stress in mammary tissue during lactation for sow health and production.

In recent decades, antioxidants either from nature or artificial synthesis have been widely accepted and used to improve animal production. This beneficial effect is attributed to their capacity of reactive oxygen species (ROS) detoxication to maintain dynamic redox balance (7, 8). Se is a well-known component of many enzymatic proteins participating in chemical reactions related to ROS neutralization (9). It is also commonly considered as an essential trace element for animal health (10–12) as it is involved in multiple biological functions such as reproduction (13), muscle metabolism (14–18), and immune response (19), as well as antiaging (20). Se deficiency has been shown to cause reduced animal health and performance especially during lactation in various animal species (21, 22). Instead, appropriate Se supplementation resulted in increased milk production (15–17) and milk quality (10, 17, 23, 24).

Based on those study results, various forms of Se were added in diets with the purpose to improve animal systemic redox status and consequently enhance lactating performance. In general, Se was added in animal diets in two different forms, which are inorganic salts mainly represented as sodium selenite, and organic selenium mainly represented as selenium methionine (Sel-Met) and selenium yeast (SeY). Comprehensive studies have been conducted to evaluate the relative efficiency of different types of Se on animal performance and achieved a common understanding that organic Se has an overall advantage in absorption rate (25), antioxidant capacity (10, 26–28), low toxicity (29), and animal health and performance improvement (30, 31). To date, the main gap in knowledge regarding the Se nutritional regulation is the comparison of different sources of organic and underlying mechanisms considering their wide application in both human being and animal feeding additives nowadays. This study therefore was conducted to compare the effects of two different sources (SeY and Sel-Met) of organic Se using pig mammary gland epithelial cells (PMECs) as an *in vitro* model by evaluating cell viability, gene transcriptome of selenoproteins, which are the main forms of Se in mammals, and antioxidant status, with the purpose to provide theoretical foundations for organic Se application in lactating sow diets.

## Materials and Methods

### Cell Culture

The PMECs used in this study were previously isolated and characterized from mammary glands of lactating sows in our lab and were used to evaluate the synthesis and/or transport of amino acids, fatty acids, and lactose of sows in our previous studies (32–36) as the following: the mammary gland tissue was cut into 1-mm^3^ fragments with sterile scissors and washed repeatedly with D-Hanks solution to wash away blood cells and other connective tissue. Then the tissue fragments were digested with collagenase II at 37°C and 5% CO_2_ for 1 h. The isolated cells were cultured in DMED/F12 medium supplemented with 10% fetal bovine serum, and the medium was changed every 24 h. Additionally, fluorescence-activated cell sorting (FACS) analysis of cytokeratin expression was used to verify the purity of mammary epithelial cells at more than 90%. In the present study, isolated cells were incubated at 37°C, 5% CO_2_ and cultured in a complete medium according to the formula of Jaeger et al. (37), which consists of Dulbecco's Modified Eagle Medium/Nutrient Mixture F-12 (DMEM/F12, GIBCO), 10% fetal bovine serum (FBS, PAA), 1% antibiotic/antimycotic solution (10,000 U/mL penicillin, 10 mg/mL streptomycin sulfate, 25 μg/mL amphotericin B, GIBCO, I-15240), 10 μg/mL insulin (Sigma, I 6634), and 1 μg/mL hydrocortisone (Sigma-Aldrich).

### Preparation of Selenium Compounds

The protease solution was prepared by adding 2 mg protease XIV (Sigma-Aldrich) into 0.5 mL 10 mM Tris-HCl (Sigma-Aldrich) buffer. Dissolve 40 mg selenium yeast (SeY, Nicholasville, KY) in the prepared protease solution and mix well. The samples were ultrasonic 25 s with 80% amplitude on ice and cleaned with ultra-pure water. The extraction power was 30 W and the samples were run for 15 min. The obtained sample was centrifuged at 14,000 rpm for 3 min, and the supernatant was taken. The precipitation was cleaned and mixed with ultrapure water until resuspension, and the supernatant was centrifuged again at 14,000 rpm for 3 min to obtain the supernatant. The concentration of selenium in the sample was determined by inductively coupled plasma mass spectrometry (ICP-MS) (38).

A total of 12 mg of Sel-Met were dissolved in 10 L DMED/F12 to prepare 1.2 ppm stock solution. The original solution was added to 5, 3.75, 2.5, 1.25, and 0 mL volumes of DMED/F12 to prepare the working solution at concentrations of 0, 0.3, 0.6, 0.9, and 1.2 ppm, respectively.

### Cell Viability Assay

Cell viability was tested using the CCK-8 assay (Dojindo, Japan) according to the manufacturer's instruction. Briefly, PMECs were seeded into 96-well microplates at 200 μL/well with 2 ×104 cells/mL and were cultured in complete medium at 37°C 5% CO_2_ for 48 h. Then, selenium yeast (SeY; Alltech Inc, Nicholasville, KY) and selenium methionine (Sel-Met, sigma, 3211-76-5, United States) were successively added to the 96-well plate at concentration gradients of 0, 0.3, 0.6, 0.9, and 1.2 ppm, respectively. At 24 h post-treatment 20 μL, CCK-8 was added to each well, incubated for 4 h at 37°C, and then measured using a microplate reader at the wavelength of 450 nm.

### RNA Isolation and Quantitative Real-Time PCR

PMECs were seeded into 6-well plates at 2 mL/well with 5 ×10^4^ cells/mL and were cultured in complete medium at 37°C, 5% CO_2_ for 48 h. Then, the cells were treated with various levels of SeY and Sel-Met (0, 0.3, 0.6, 0.9, or 1.2 ppm) for 24 h. After that, total RNA was extracted from PMECs using TRIZOL (Invitrogen catalog no. 15596–026), according to the manufacturer's instructions. The quality and quantity of RNA were analyzed by Agilent Bioanalyzer 2100 using an RNA 6000 Labchip kit. Potential DNA contamination of the extraction was eliminated using the DNA-free kit (Ambion, catalog no. AM1906), and the RNA quality was verified by both agarose gel (1%) electrophoresis, and spectrometry (A260/A280). First-strand cDNA synthesis was performed by using a Prime Script RT reagent kit with gDNA eraser (Takara, Dalian, China). The cDNA was synthesized from 1 μg total RNA using Super Script III reverse transcriptase according to the manufacturer's instructions. The mRNA levels of 25 selenoprotein genes were analyzed by qPCR using the SYBR Green PCR Master Mix according to the manufacturer's instructions (Cat# RR047A, TakaRa). Primers for the 25 selenoprotein genes were referenced from the study of Zhao et al. (39) (Table 1), and primer for the β-actin gene (Actb) was from our previous study (40). The 2^−ΔΔCt^ method (41) was used for the quantification with the β-actin gene as a reference gene, and the relative abundance was normalized to the control.

**Table 1:** Primers used for RT-PCR.

**Gene**	**Accession number**	**Primer pairs (5' to 3' direction)**
DIO1	AY533206	F: CATGGCCAAGAACCCTCACT
		R: CCAGAAATACTGGGCACTGAAGA
DIO2	AY533207	F: CGCTGCATCTGGAAGAGCTT
		R: TGGAATTGGGTGCATCTTCA
DIO3	AY533208	F: TGAAGTGGAGCTCAACAGTGATG
		R: TGTCGTCAGACACGCAGATAGG
GPX1	AF532927	F: GATGCCACTGCCCTCATGA
		R: TCGAAGTTCCATGCGATGTC
GPX2	DQ898282	F: AGAATGTGGCCTCGCTCTGA
		R: GGCATTGCAGCTCGTTGAG
GPX4	NM_214407	F: TGAGGCAAGACGGAGGTAAACT
		R: TCCGTAAACCACACTCAGCATATC
GPX6	NM_001137607	F: GAGCTGAAGCCTTTTGGTGTAGTT
		R: CTTTGCTGGTTCTTGTTTTCCA
SELH	HM018602	F: TGGTGGAGGAGCTGAAGAAGTAC
		R: CGTCATAAATGCTCCAACATCAC
SELR	NT_033777.3	F: GAACCACTTTGAGCCAGGTGTCTAC
		R: GCCTTTAGGGATGAACTTCAGGGAAC
SELW	NM_213977	F: CACCCCTGTCTCCCTGCAT
		R: GAGCAGGATCACCCCAAACA
SPS2	BM489698.1	F: CGTTGGGTATCGGAACTGAC
		R: CGTCCACCAGAGGGTAGAAA
SELI	EST	F: GATGGTGTGGATGGAAAGCAA
		R: GCCATGGTCAAAGAGTTCTCCTA
SELK	DQ372075	F: CAGGAAACCCCCCTAGAAGAA
		R: CTCATCCACCGGCCATTG
SELM	FJ968780	F: CAGCTGAATCGCCTCAAAGAG
		R: GAGATGTTTCATGACCAGGTTGTG
SELN	EF113595	F: ACCTGGTCCCTGGTGAAAGAG
		R: AGGCCAGCCAGCTTCTTGT
TXNRD1	AF537300	F: GATTTAACAAGCGGGTCATGGT
		R: CAACCTACATTCACACACGTTCCT
TXNRD2	GU181287	F: TCTTGAAAGGCGGAAAAGAGAT
		R: TCGGTCGCCCTCCAGTAG
TXNRD3	BX918808	F: GTGCCCTACGTTTATGCTGTTG
		R: TCCGAGCCACCAGCTTTG

*ACTB, beta actin; DIO, iodothyronine deiodinase; GPX, glutathione peroxidase; SELH, I, K, M, selenoproteins H, I, K, M; SEPHS2 (SPS2), selenophosphate synthetase 2; SELW, selenoprotein W; TXNRD, thioredoxin reductase*.

### Western Blot Analysis

PMECs were seeded into 6-well plates at 2 mL/well with 5 ×10^4^ cells/mL and were cultured in complete medium at 37°C 5% CO_2_ for 48 h. Then, the cells were treated with 0.6 ppm SeY and Sel-Met for 24 h. After that, cells were collected and homogenized in RIPA lysis buffer (Beyotime, Nanjing, China). The Western blot analysis was done according to the procedures described in our previous study (33). In general, the same number of samples were electrophoretic ally separated and transferred to PVDF membrane, which was sealed at room temperature with 5% skim milk for 2 h. The primary antibody of the target protein was added and incubated at 4°C for 12 h, then the secondary antibody was incubated for 1.5 h, and the chemiluminescence reaction was carried out. The primary antibody was diluted according to the instructions: GPX1 antibody (1:1000, 3206, Cell Signaling Technology, United States), TrxR3 antibody (1:1000, 19517-1-AP, Proteintech, United States), JNK (1:1000, 66210-1-lg, Proteintech, United States), P-JNK (1:1000, 80024-1-RR, Proteintech, United States), Bax (1:1000, 50059-2-lg, Proteintech, United States), Bcl-2 (1:2000, 60178-1-lg, Proteintech, United States), P-p38 (1:1000, 4511, Cell Signaling Technology, United States), p38 (1:1000, 8690, Cell Signaling Technology, United States), Caspase 3 (1:1000, 9662, Cell Signaling Technology, United States), and β-actin (1:2000, bs-0061R, Bioss, China).

### Antioxidant Enzymes Assay

PMECs were seeded into 6-well plates at 2 mL/well with 5 ×10^4^ cells/mL and were cultured in complete medium at 37°C 5% CO_2_ for 48 h. Then, the cells were treated with 0.6 ppm SeY and Sel-Met for 24 h. The collected cells were cleaned with PBS for two times and centrifuged to retain cell precipitation. The cells were suspended with 0.5 mL isotonic PBS buffer, and the broken cells were ground in the grinding machine to obtain PMECs cell suspension, which was used to detect the content of superoxide dismutase (SOD), malondialdehyde (MDA), catalase (CAT), and the total antioxidant capacity (T-AOC) of cells. The antioxidant capacity of cells was determined using the kit of Nanjing JianCheng Institute of Biological Engineering, and the detection method was according to the instructions of the kit.

### Statistical Analysis

The data of CCK-8, PCR, Western blot, and antioxidant status were analyzed by one-way ANOVA, LSD multiple comparison, and Pearson correlation analysis using SPSS 22.0 software in the present study. The establishment of PCA and heatmap models was performed on the Tutools platform (https://www.cloudtutu.com). The *P*-value < 0.05 was used as the criterion to judge the significance of the difference.

## Results

### Appropriate Se Supplementation Promoted PMEC Cell Viability

**Figure 1:**
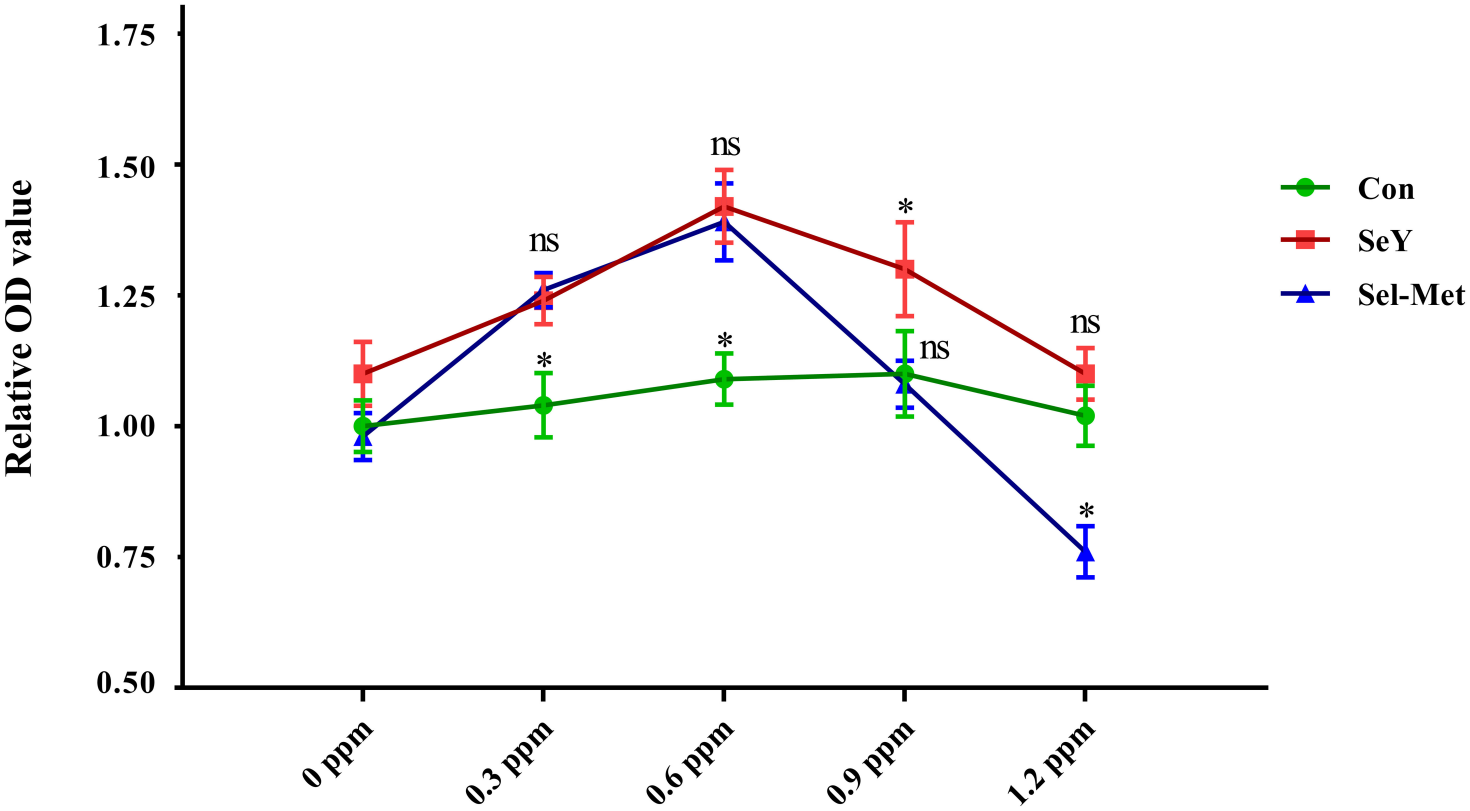
Effects of SeY and Sel-Met supplementation on cell viability in PMECs. Cells were incubated with different concentrations of SeY and Sel-Met (0, 0.3, 0.6, 0.9, and 1.2 ppm) for 24 h, respectively. Cell viability was analyzed using the CCK-8 assay. Data are expressed as mean ± SEM (*n* = 12). Superscript * indicates significant (*P* < 0.05).

As shown in Figure 1, compared with the control group, both 0.3 and 0.6 ppm SeY and Sel-Met resulted in higher cell viability (*P* < 0.05). At the increased concentrations of 0.9 ppm SeY still led to greater cell viability, but Sel-Met had no affect (*P* > 0.05), while at 1.2 ppm SeY was not different from the control and Sel-Met treated cells had lower viability (*P* < 0.05).

### Se Supplementation Altered Selenoprotein Gene Profile of PMECs

**Figure 2:**
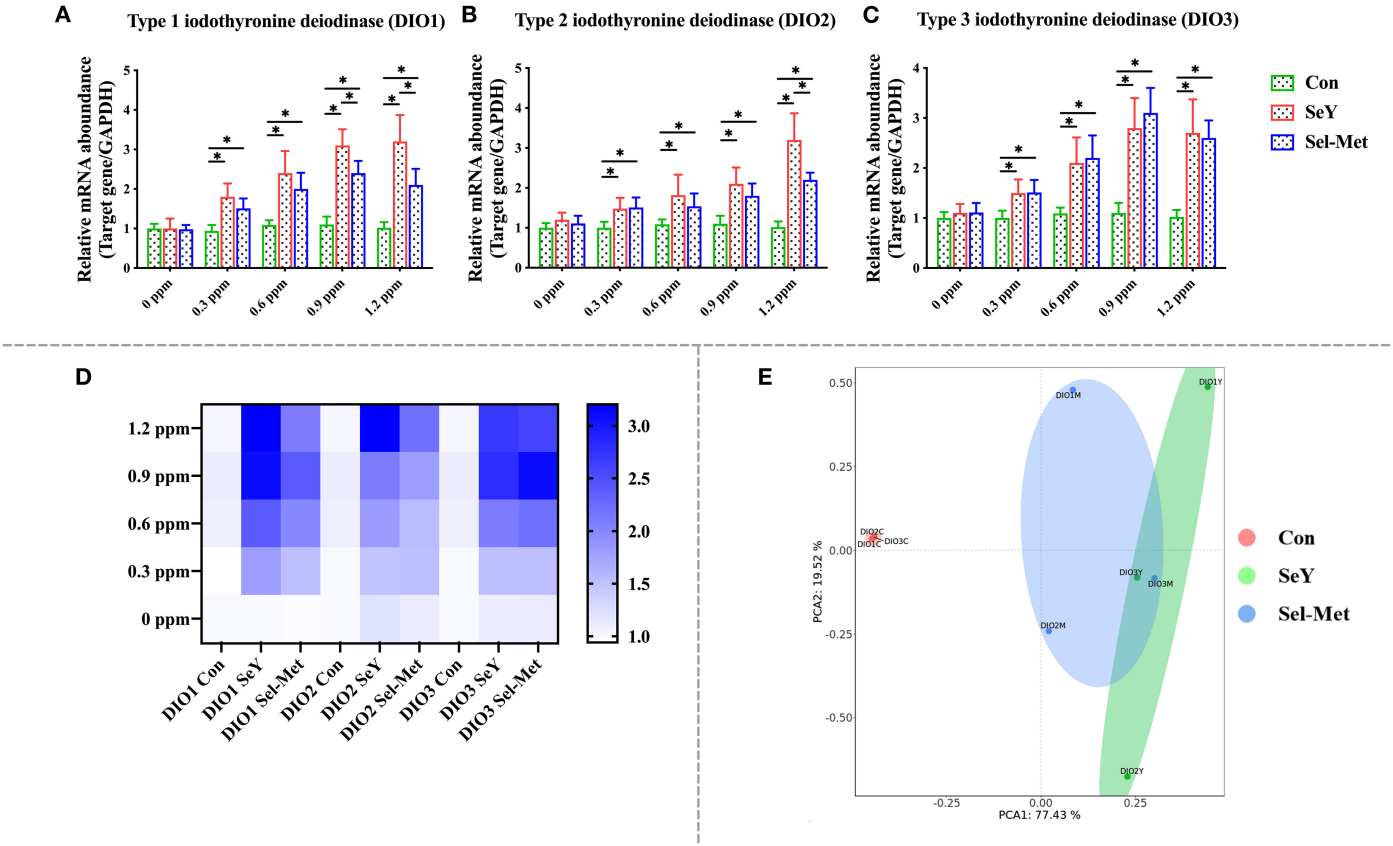
Supplement SeY and Sel-Met to mRNA expression of DIOs family in PMECs. The cells were incubated with SeY and Sel-Met at different concentrations (0, 0.3, 0.6, 0.9, and 1.2 ppm) for 24 h, respectively, and then collected to detect mRNA expression. Data were expressed as mean ± SEM (*n* = 6), and different superscripts * indicate statistically significant differences (*P* < 0.05). **(A–C)** Are the mRNA expression level of DIOs detected by qPCR. **(D)** Heat map comparison of DIOs mRNA levels. **(E)** PCA score plot results compared the mRNA expression of the DIOs family among the three groups.

Figure 2 describes the mRNA expression of DIOs family in PMECs cells after SeY and Sel-Met treatment. The gene expression of DIO1, DIO2, and DIO3 mRNA were significantly up-regulated in 0.3 and 0.6 ppm of two different sources of organic Se treated cells (Figures 2A–C) (*P* < 0.05), and there was no significant difference between SeY and Sel-Met. When Se concentration was increased up to 0.9 and 1.2 ppm, the supplementation of both forms of Se lead to higher mRNA expression of DIO1, DIO2, and DIO3 (*P* < 0.05), but SeY groups showed higher mRNA expression levels of DIO1 and DIO2 compared with those with Sel-Met at the same concentration (*P* < 0.05). The PCA (Figure 2E) diagram can represent the difference between different groups found. The farther the spatial distance between different samples is, the greater the data difference between them is. The non-overlapping area between SeY group and Sel-Met group was larger than the overlapping area, indicating that there was a significant difference in the addition effect of the two groups on the mRNA expression of DIOs family. Taken together, these data suggest that SeY is more effective in increasing DIOs expression than addition of Sel-Met.

**Figure 3:**
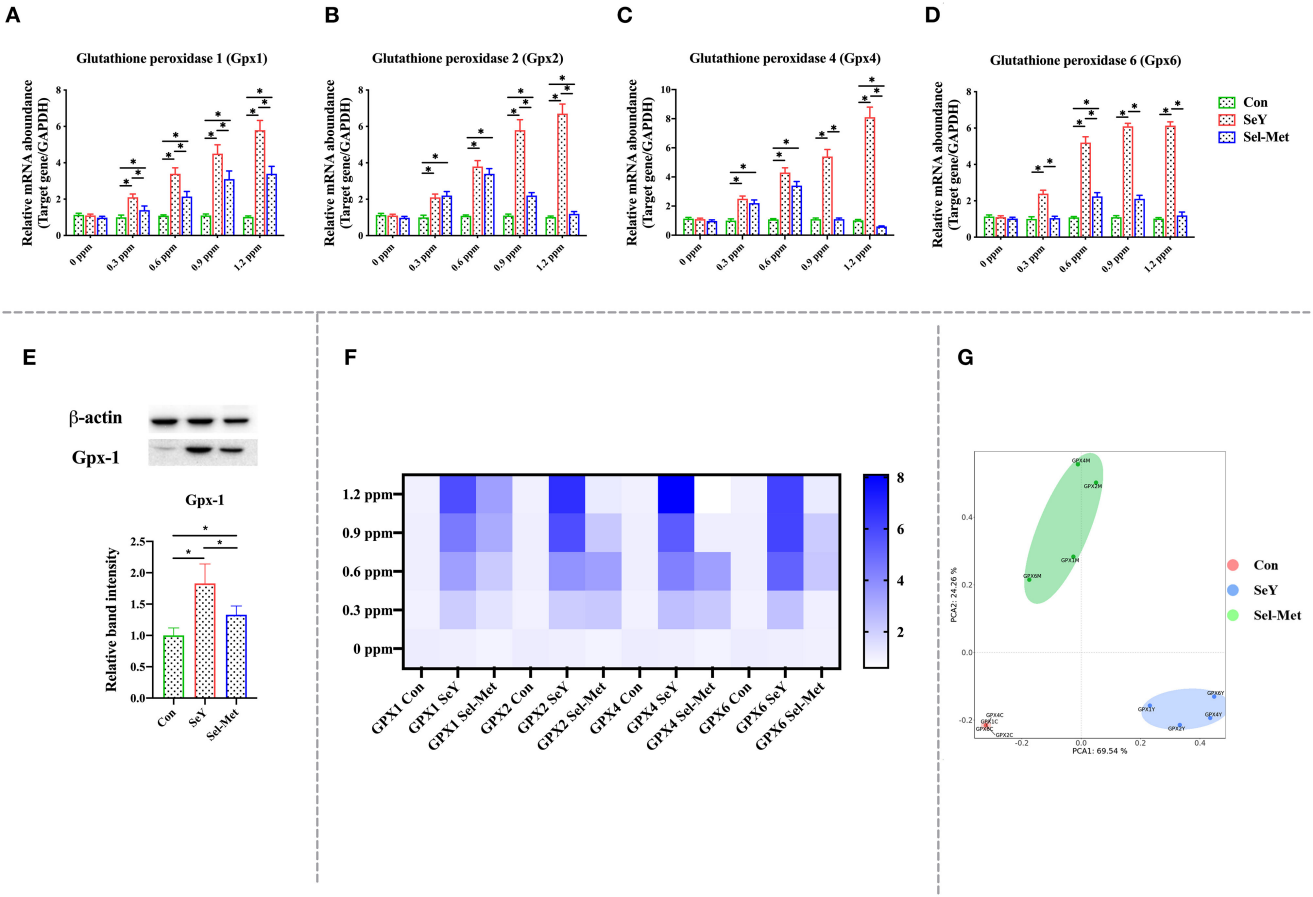
Effects of SeY and Sel-Met addition on the mRNA expression of glutathione peroxidase family in PMECs. The cells were incubated with different concentrations of SeY and Sel-Met (0, 0.3, 0.6, 0.9, and 1.2 ppm) for 24 h, respectively, and then collected for detection. Data were expressed as mean ± SEM (*n* = 6), and * indicated statistically significant difference (*P* < 0.05). **(A–D)** mRNA expression levels of GPXs family were detected by qPCR. **(E)** The relative protein levels of GPX1 were analyzed by Western blotting. **(F)** Heat map comparison of mRNA expression levels of GPXs. **(G)** PCA score plot results compared the mRNA expression of GPXs between the three groups.

Figure 3 illustrates the changes of GPX family gene and protein expression after SeY and Sel-Met treatment. The mRNA expression levels of GPX1, GPX2, GPX4, and GPX6 in SeY group were significantly higher than those in the control group (Figures 3A–D) (*P* < 0.05). For Sel-Met group, the GPX1 mRNA expression level of different supplemental levels was higher than that of the control group (*P* < 0.05), but lower than that of the SeY group (*P* < 0.05). The mRNA expression levels of GPX2 and GPX4 were similar in the Sel-Met group and the SeY group at 0.3 and 0.6 ppm. In particular, 1.2 ppm Sel-Met inhibited GPX4 expression in PMECs (*P* < 0.05). At 0.6 ppm, GPX6 mRNA level in the Sel-Met group was higher than that in the control group, except that the mRNA expression levels of other supplemental levels were the same as that in the control group (*P* < 0.05). Combined with heat map (Figure 3F) observation, it can be concluded that the SeY group has a much better promotional effect on the mRNA expression level of the GPX family in PMECs than the Sel-Met group. By PCA figure can be seen that the SeY and Sel-Met groups have obvious distinctions (Figure 3G) that both in different addition amounts of GPXs mRNA expression of family has a significant difference. This result was also verified by Western blot assay (Figure 3E) (*P* < 0.05).

**Figure 4:**
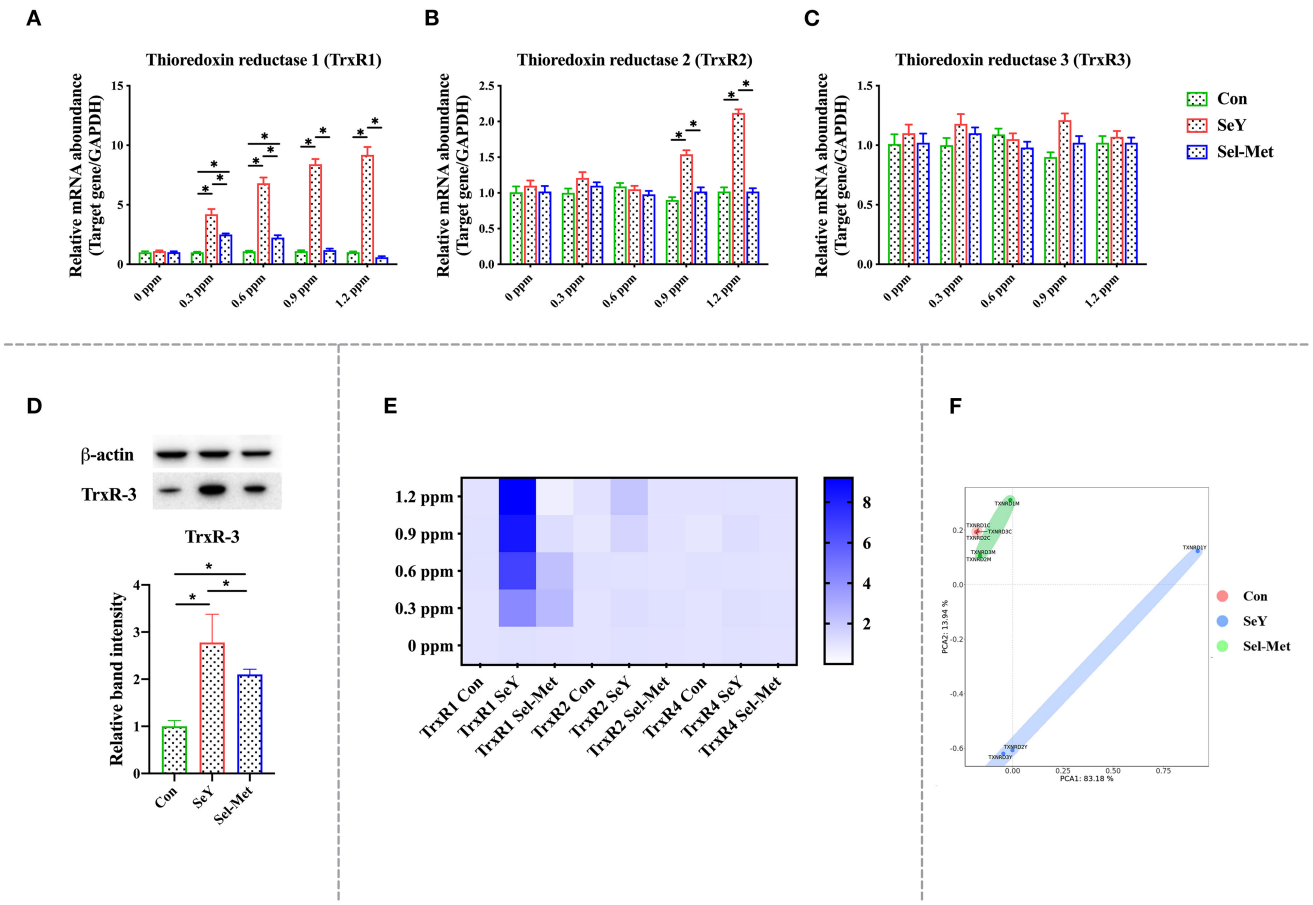
Effects of SeY and Sel-Met addition on mRNA expression of TrxRs family in pMECs. The cells were incubated with different concentrations of SeY and Sel-Met (0, 0.3, 0.6, 0.9, and 1.2 ppm) for 24 h, respectively, and then collected for detection. Data were expressed as mean ± SEM (*n* = 6), and * indicated statistically significant difference (*P* < 0.05). **(A–C)** mRNA expression level of TrxRs family was detected by qPCR. **(D)** The relative protein levels of TrxR3 were analyzed by Western blotting. **(E)** Heat map comparison of TrxRs mRNA expression levels. **(F)** PCA score plot results compared the mRNA expression of the DIOs family among the three groups.

Observe Figure 4, which depicts mRNA and protein expression of the thioredoxin reductase family (TrxR). The addition of SeY and Sel-Met at different concentrations had no effect on TrxR3 mRNA expression (*P* > 0.05), but TrxR1 and TrxR2 mRNA expression were different. TrxR1 mRNA expression in SeY groups was significantly higher than that in the control group (*P* < 0.05). Sel-Met significantly increased TrxR1 mRNA expression at 0.3 and 0.6 ppm, while it was similar to the control group at 0.9 and 1.2 ppm. TrxR2 mRNA expression was significantly increased in 0.9 and 1.2 ppm SeY groups (*P* < 0.05), while there was no significant change in the Sel-Met group (Figures 4A–C). In the PCA diagram, Sel-Met and SeY groups did not overlap at all, and the above results indicated that there was a significant difference in addition effect between the two groups (Figure 4F). The heat map clearly shows the addition effect of SeY, which is significantly better than Sel-Met (Figure 4E). Western blot detection showed that TxrR3 protein expression in both Se supplemental groups was better than that in the control group (Figure 4D), but the effect of SeY addition was more significant (*P* < 0.05).

**Figure 5:**
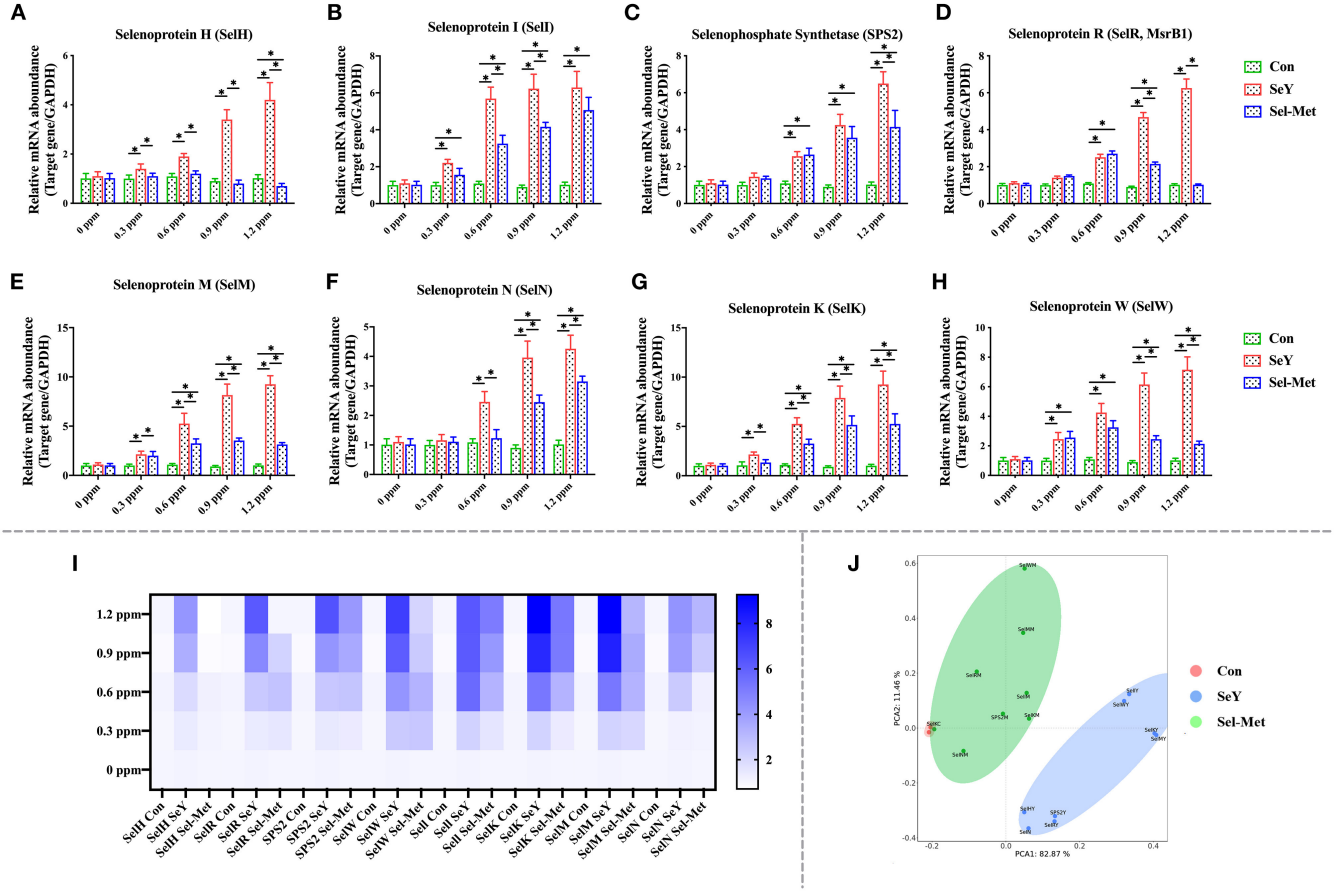
Effects of SeY and Sel-Met addition on mRNA expression of SelH, Sell, SPS2, SelM, SelN, SelK, SelR, and SelW in PMECs. The cells were incubated with different concentrations of SeY and SEL-Met (0, 0.3, 0.6, 0.9, and 1.2 ppm) for 24 h, respectively, and then collected for detection. Data were expressed as mean ± SEM (*n* = 6), and * indicated statistically significant difference (*P* < 0.05). **(A–H)** qPCR was used to detect the mRNA expression levels of SelH, Sell, and other selenium proteins. **(I)** Heat map comparison of mRNA expression levels of SelH, Sell, and eight other selenoproteins. **(J)** PCA score plot results the mRNA expression levels of SelH, Sell, SPS2, SelM, SelN, SelK, SelR, and SelW were compared among the three groups.

As presented in Figure 5, the mRNA expression of SeIH was greater with all SeY concentration levels compared with control. Increasing concentrations of SeY from 0.3 to 1.2 ppm significantly and consistently elevated the mRNA expression of SeIR, SeIW, and SPS2 (Figures 5C,D,H) (*P* < 0.05). However, Sel-Met did not affect the mRNA expression of SeIH (Figure 5A) (*P* > 0.05) from 0.3 to 0.9 ppm, and lowered (*P* < 0.05) the mRNA expression of SeIH when added at 1.2 ppm. The mRNA expression of SelR, SelW, and SPS2 increased significantly after adding 0.6 and 0.9 ppm Sel-Met (Figures 5C,D,H) (*P* < 0.05). At 1.2 ppm, Sel-Met increased the mRNA expression of SelW and SPS2 (*P* < 0.05) but had no effect on SelR (*P* > 0.05). At the same amount of addition, the expression of SelH, SelR, SelW, SPS2 in Sel-Met cells was the same as that of SeY or lower than that of SeY (*P* < 0.05). With the increase of SeY and Sel-Met levels from 0.3 to 1.2 ppm, the mRNA expression levels of Sell, SelK, and SelM were higher than those in the control group (Figures 5B,E,G). SelN mRNA expression increased with the increase of concentration except 0.3 ppm (Figure 5F) (*P* < 0.05). The expression of SelN at 0.9 and 1.2 ppm was significantly higher than that in the control group. Except for the same expression of Sell at 1.2 ppm, the effect of the SeY group was better than the Sel-Met group (*P* < 0.05). In the PCA diagram (Figure 5J), the Sel-Met group overlapped with the control group and was clearly distinguished from the SeY group. As can be seen, the difference between the Sel-Met group and the control group was small, but there was significant difference between the Sel-Met group and the SeY group.

### Se Supplementation Promoted Cellular Antioxidative Capacity

**Figure 6:**
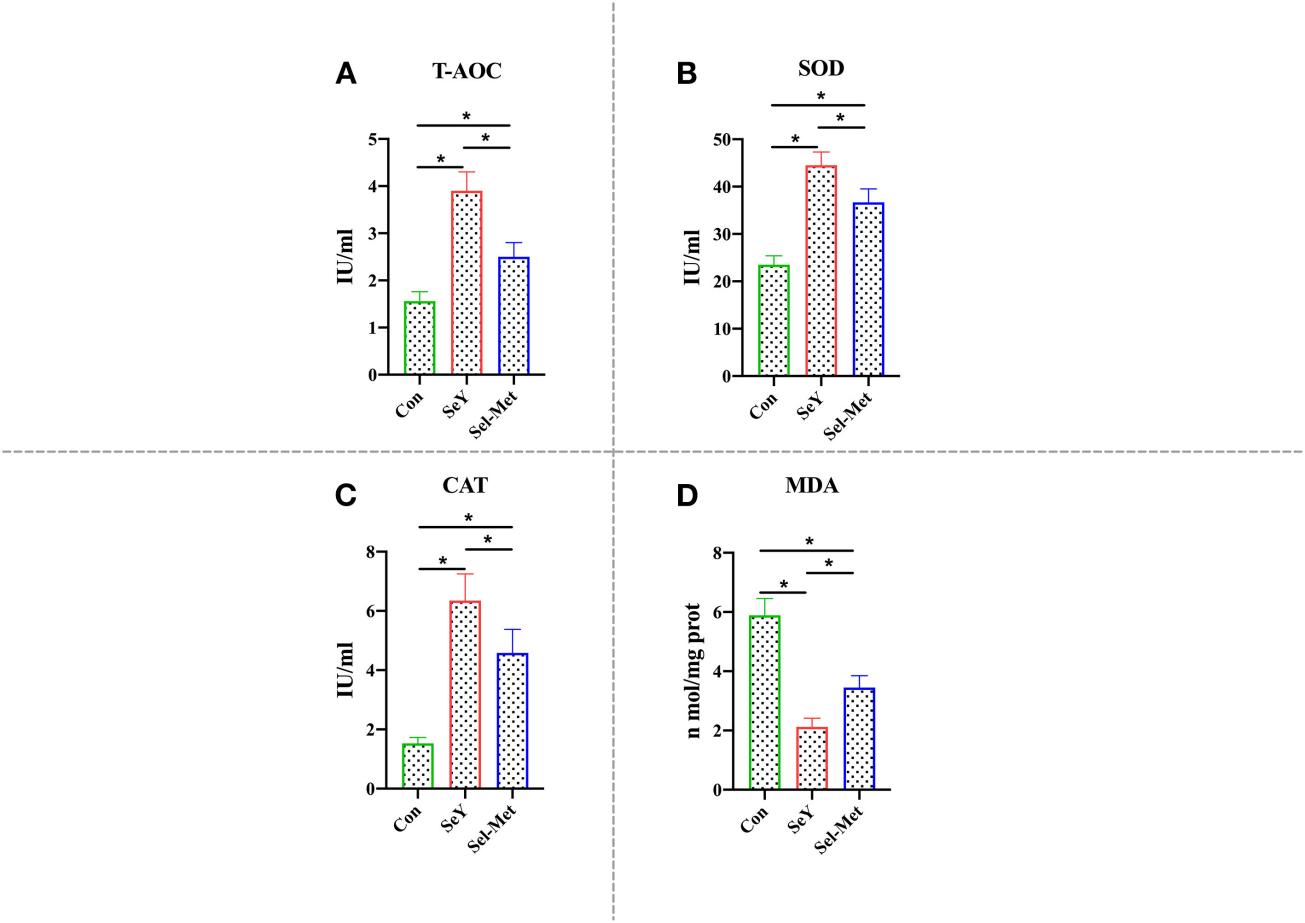
Effects of SeY and Sel-Met supplementation on **(A)** T-AOC, **(B)** SOD, **(C)** CAT, and **(D)** MAD in PMECs. Cells were incubated for 24 h with 0.6 ppm SeY and Sel-Met, and then were collected for T-AOC, SOD, CAT, and MAD activity analysis. Data are expressed as mean ± SEM (*n* = 6). *Indicates that the difference was statistically significant (*P* < 0.05).

As can be seen in Figure 6, it is clear that T-AOC, SOD, and CAT levels were significantly higher with either Se source (*P* < 0.05) and compared with Sel-Met, the groups with SeY addition had significantly higher (*P* < 0.05) T-AOC, SOD, and CAT. In contrast, the MDA levels are significantly lower (*P* < 0.05) for both groups supplemented with selenium addition compared with the control. Furthermore, the MDA levels are significantly higher (*P* < 0.05) with Sel-Met compared to SeY.

### JNK/p38 Pathway Was Inhibited by Supplementation of Se

**Figure 7:**
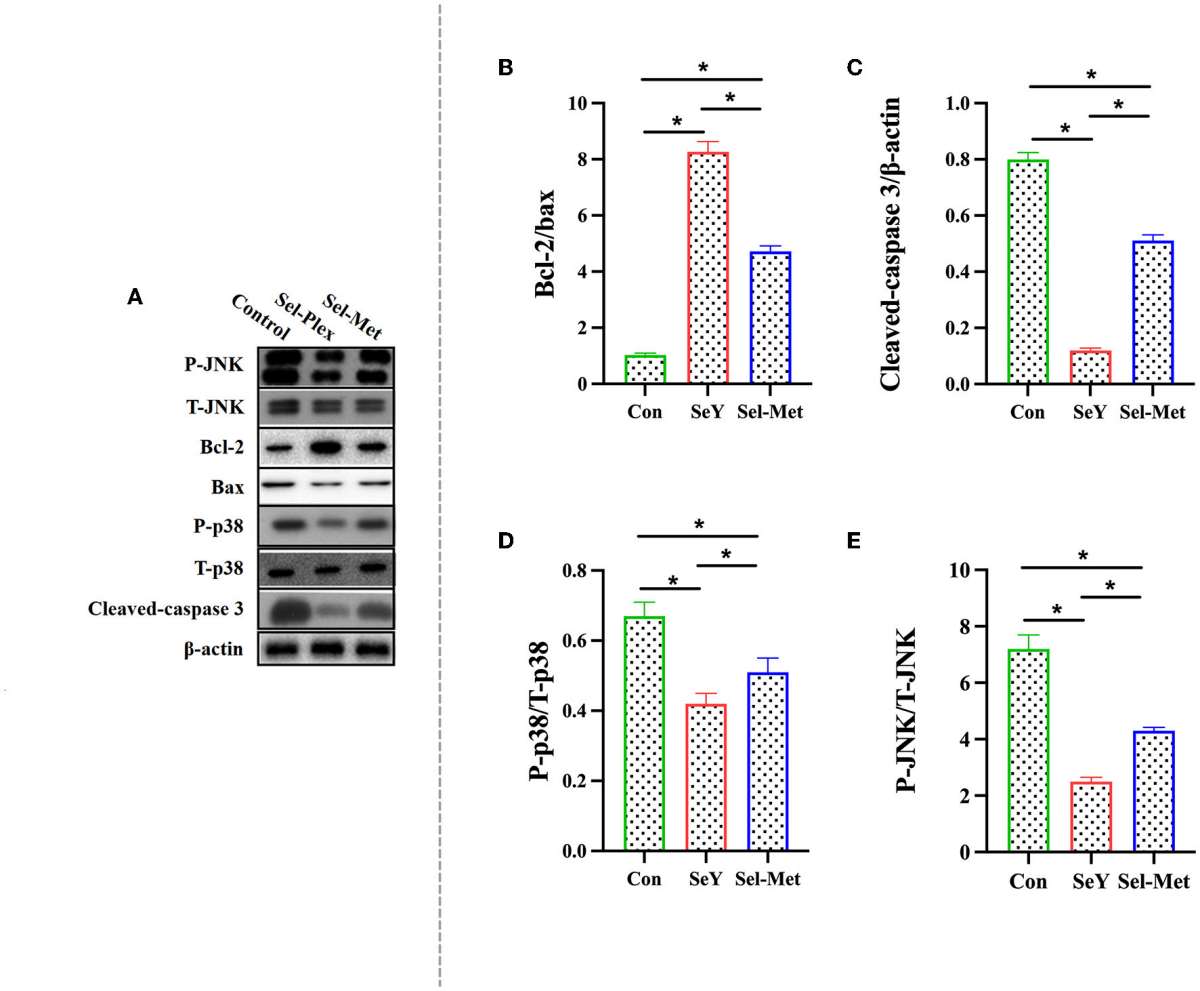
Effects of SeY and Sel-Met supplementation on **(A)** is the original band of Western Blot, **(B)** Bcl-2/Bax, **(C)** Caspase-3, **(D)** P-p38/T-p38, and **(E)** P-JNK/T-JNK protein expression in PMECs. Cells were incubated for 24 h with 0.6 ppm SeY and Sel-Met and then were collected for the determination of protein expression. Data are expressed as mean ± SEM (*n* = 3). *Indicates that the difference was statistically significant (*P* < 0.05).

Figure 7 showed that the ratio of p-JNK/JNK and p-p38/p38, and the abundance of cleaved-caspase3 were significantly lower in the SeY group and Sel-Met groups compared to the control, with SeY being lower (*P* < 0.05) than Sel-Met. The abundance of Bcl-2 was significantly higher (*P* < 0.05) with both Se sources compared to the control, with SeY being higher (*P* < 0.05) than Sel-Met.

**Figure 8:**
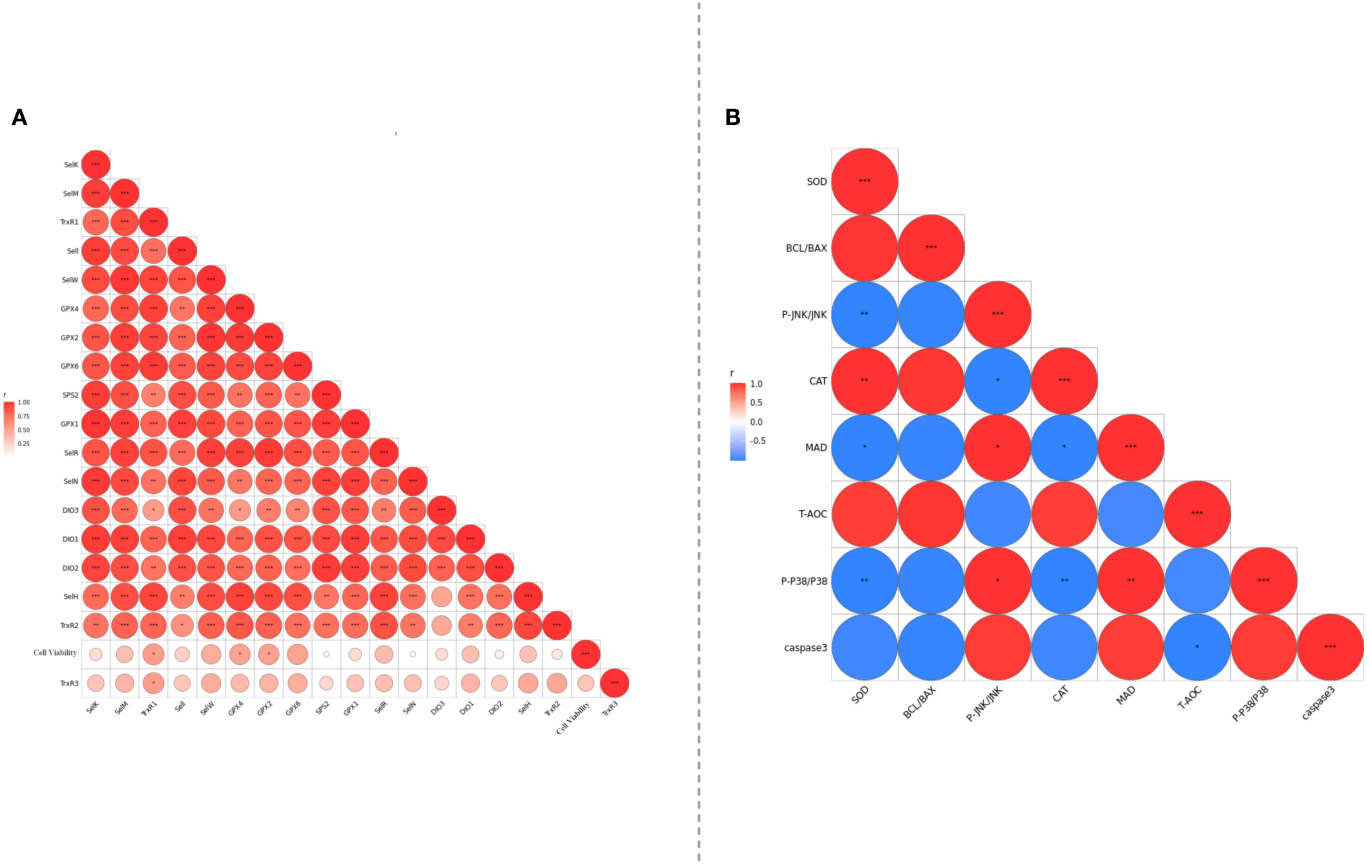
**(A)** Analyzed the correlation between selenoprotein mRNA expression levels of PMCES cultured with different additives and different dosage. **(B)** The degree of correlation between PMCEs related indicators of antioxidant and apoptosis was analyzed. The area size and color of the circle in the figure represent the correlation, *0.01 < *P* <0.05; **0.001< *P* ≤ 0.01; ***P ≤ 0.001.

To further explore the interaction between selenoproteins, correlation analysis was conducted. As shown in Figure 8, the expressions of various selenoproteins except TrxR3 were obviously positively correlated (Figure 8A). There was a negative correlation between apotheosis index and antioxidant index (Figure 8B).

## Discussion

Se has been shown to have beneficial effects in cell proliferation and anti-apoptosis process due to its antioxidative properties in previous studies (42). Zeng reported that Se deficiency resulted in a decreased number of G1 phase cells that corresponded to increased numbers of G2 and sub-G1 phase cells, while 0.25 μmol/L Sel-Met addition significantly enhanced the expression of cell cycle-related genes and reversed this cell growth trend (43). In addition, several sources of Se including Na_2_SeO_3_, Sel-Met, and selenite containing Se compounds all showed positive effects on cell viability in various cells including human immature dendritic cells, human lens epithelial cells (44), chondrocyte ATDC5 cells (42), fibroblast HT1080 cells, and primary porcine macrophages cells (45, 46). Thus, it is not unexpected that reasonable levels of both SeY and Sel-Met supplementation improved the cell viability of PMECs in the present study (Figure 1). Interestingly, we found inconsistent trends between the two groups of cells when treated with 0.9 and 1.2 ppm, with SeY having no effect on cell viability in the 1.2 ppm range, while Sel-Met began to show inhibition. In previous studies, organic Se has been shown less toxic due to its higher retention in animal tissues and lower concentration levels in plasma compared with inorganic Se (47), but there is a lack of data regarding negative effect of too much excessive organic Se and comparison of toxic effects of different sources of organic Se. The result of the present study suggested that excessive Sel-Met addition might be toxic to cells and potentially induce cellular damage. The absence of impaired cell growth caused by high concentration of SeY in the current study implied that cells probably have higher tolerance to SeY than Sel-Met at the super nutritional level, which mechanism should be paid more attention to in the future.

Se acts as an antioxidant regulating cellular redox balance mainly in the form of selenoprotein including the glutathione peroxidase family (GPXs), thioredoxin reductase family (TrxR), and deiodinase family (DIOs), as well as SelH, SelI, SelM, SelK, etc. (48, 49). The GPX family is a group of antioxidant enzymes with Se as the active component to decompose peroxides into non-toxic hydroxyl compounds thus preventing peroxide from peroxidation of the cell membrane lipids (50–52). In cells, ROS could be neutralized by glutathione under catalyzation of GPX to generate oxidized glutathione (GSSH), and then GR catalyzes the regeneration of GSH from GSSH, which recycle of GSH producing were defined as glutathione system playing important roles in cellular redox maintenance. Similar to the glutathione system, thioredoxin (Trx) and TrxR make up the thioredoxin system and were involved in multiple ROS scavenging processes through reversible Trx oxidation/reduction reaction catalyzed by TrxR (53, 54). Current research mainly focuses on TrxR1 and TrxR2, both of which play a key role in protecting cells from oxidative stress damage, while TrxR3 does not play a significant role in antioxidant effects. In studies on antioxidants, most of the supplements had an effect on the mRNA expression of TrxR1 and TrxR2, but had no significant change in the expression of TrxR3 (55, 56). It was well-established that Se status within cells and tissues is a key regulator of selenoproteins expression both *in vivo* and *in vitro* (57). For instance, Se supplementation in cell medium greatly increased the mRNA abundance of GPX1, SELH, SELN, SELP, and SELW in ATDC5 cells, and GPX1, SELH, SELN, SELP, SELW, and GPX3 in C28/I2 cells, while decreased the mRNA abundance of SPS2 and SELO in ATDC5 cells, and SPS2, SELO, TRXR2 in C28/I2 cells (58). Stolwijk et al. (59) showed that the activities of GPX1 and GPX4 were significantly up-regulated in the exponentially growing cells cultured in the medium supplemented with 200 nM Seleno-L-methionine. Similarly, Doroshow et al. (60) also found that 30 nM sodium selenite addition to the cell culture medium significantly increased the activity of GPX using NCI/ADR-RES cancer cells as an *in vitro* model. In addition, dietary Se supplementation also has been reported to upregulate selenoprotein transcriptome in chicken embryonic neurons, liver, and muscle (61). Furthermore, Se deficiency disease has been shown to be related to decreased mRNA expression of several selenoprotein genes (GPX1, GPX4, SEPW1, SEPN1, SEPP1, SELO, and SELK) in liver and muscle (62). Dietary Se deficiency also has been reported to significantly reduce the mRNA expression levels of DIO, GPX3, and TXNRD2 in the muscle of broilers (63). In the present study, we compared the effects of two different organic Se to gene expression of selenoproteins and results showed that both SeY and Sel-Met addition in cell medium increased gene expression of most selenoproteins including GPX1, GPX2, GPX4, GPX6, and TrxR1, TrxR2, which is consistent with previous findings. In addition, in terms of protein expression, the addition of two selenium sources significantly increased the protein expression of GPX1 and TrxR3. We found that SeY had a better beneficial effect than Sel-Met at the same level of addition, combining gene and protein experimental results. To our best knowledge, this result is the first time to show the different impact of different sources of organic Se on global selenopreoteins using an *in vitro* model.

SeY, not as Sel-Met, is a complex of multiple macronutrients including Sel-Met as the main component, unknown forms of organic Se, and other bioactive elements originally from yeast. Accumulating evidence showed that selenium could interact with kinds of elements such as vitamin C (64), zinc (65), cadmium (66) and others to participate in redox status regulation and the synergism of multiple components resulted in higher efficiency than single one element. Salah et al. reported that co-treatment of Se and zinc more efficiently attenuated the oxidative stress induced by NiCl_2_ in pregnant Wistar rats than Se/Zinc supplementation (67). Chen et al. assessed the effect of vitamin E, Se, and co-treatment of vitamin E and Se on sow performance and they also overserved synergistic effect of vitamin E and Se (23). We did not find much literature related to Se synergism with other substrates using animal models due to the limit in this field, but we presume that the synergistic effect of multiple components in SeY might make the main contribution to its higher efficiency in the present study.

It is well-known that Se can repair damage caused by oxidative stress and promote cellular antioxidant capacity (68). Liu et al. showed that dietary selenium-enriched yeast apparently promoted piglet antioxidant status and attenuated oxidative stress-induced growth retardation (69). An investigation conducted on chickens also reported that Se administration significantly relieved oxidative stress and testicular toxicity induced by lead exposure, associated with increased activity of antioxidant enzymes, and reduced inflammatory response (70). To further compare the antioxidant effect of various sources of Se on PMECs, we tested the contents of SOD, MDA, CAT, and T-AOC, which are key makers (T-AOC, SOD, CAT, and MDA) of antioxidant status in PMCEs cells. It is clear that both organic Se have a positive effect on the antioxidant status of PMECs with increased T-AOC, SOD, and CAT, while decreased MDA, which result is consistent with previous findings that Se improved levels of antioxidant markers and ameliorated the damage caused by LPS-induced in bovine mammary epithelial cells (71). Similar to the gene expression of selenoproteins, we also observed higher antioxidative capacity of SeY compared with the same supplementation of Sel-Met, which result might be attributed to the synergistic benefit.

Oxidative stress is a result of imbalance between accumulation of ROS and the ability to get rid of it in cells and tissues (72). Severe oxidant stress induced by excessive ROS has been shown to trigger cell apoptosis associated with damaged DNA, oxidation of polyunsaturated fatty acids and amino acids, as well as mitochondrial dysfunction (73). Previous studies reported that Se status in cells and tissues plays a key role in the regulation of apoptosis process and dietary supplementation of Se might be a potential strategy to promote animal health by reducing apoptosis induced by oxidant stress (74). Wang et al. demonstrated that Se deficiency in cell medium lead to increased intracellular ROS content and activated apoptotic process *via* caspase-3 signal pathway in human uterine smooth muscle cells (75). On the other hand, Se supplementation significantly attenuated the damage and apoptosis caused by bisphenol Se in mice (76). In cells, once apoptosis was initiated, active caspase-8 directly cleaves pro-caspase-3 and triggers downstream events of apoptosis signaling. Here we found that both SeY and Sel-Met supplementation significantly reduced protein expression of cleaved caspase-3 implying that Se addition protected cells from apoptosis, which results were further confirmed by decreased ratio of Bax/Bcl-2, another most used parameter for cellular apoptosis.

A growing body of literature has shown that p38 and c-Jun-N-terminal kinase (JNK), both of which belong to mitogen-activated protein kinases (MAPKs) are involved in the mediation of cellular apoptosis induced by oxidative stress (77). Yeo et al. found that sodium selenite exerted a profound preventive effect on cell apoptosis *via* inhibition of p38 mitogen-activated protein kinase, pSAPK/JNK, and Bax activation in *in vivo* and *in vitro* rat spinal cord injury models (78). A study conducted by Wang et al. indicated that Se deficiency induced cell apoptosis by increasing gene expression of p-P38 and p-JNK in human uterine smooth muscle cells (75). In the present study, similarly we also observed the changed expression of key protein in p38/JNK signaling pathway among those cells with different treatment implying its possible involvement in the modulation of Se on PMECs apoptotic process. The lower ratio of p-p38/p38 and p-JNK/JNK in the groups treated with SeY compared with Sel-Met was consistent with other findings in the current study that SeY has more efficient effect to prevent cells from oxidant stress and apoptotic damage due to syngenetic benefit of multiple elements included in SeY.

## Conclusion

Based on the results of the present study, we summarized that both SeY and Sel-Met promoted cell viability and attenuated cell apoptosis by regulating the selenoprotein expression and antioxidative capacity *via* p38/JNK signaling pathway in PMECs, but SeY comprehensively exhibited more efficient benefit than that of Sel-Met. These findings provide a reference for scientific utilization of organic Se and enrich the theoretical research for further investigations exploring more potential nutritional regulation of Se in humans being healthy and animal production.

## Data Availability Statement

The raw data supporting the conclusions of this article will be made available by the authors, without undue reservation.

## Ethics Statement

The animal study was reviewed and approved by South China Agricultural University.

## Author Contributions

FC, SZ, and WG designed the experiment and conducted the final proofreading. CW drafted the manuscript, carried out the experiment, and performed the analysis of all data. All authors have read and agreed to the published version of the manuscript.

## Funding

This research was funded by the General Program of the Natural Science Foundation of Guangdong Province (2021A1515010944).

## Conflict of Interest

The authors declare that the research was conducted in the absence of any commercial or financial relationships that could be construed as a potential conflict of interest.

## Publisher's Note

All claims expressed in this article are solely those of the authors and do not necessarily represent those of their affiliated organizations, or those of the publisher, the editors and the reviewers. Any product that may be evaluated in this article, or claim that may be made by its manufacturer, is not guaranteed or endorsed by the publisher.

## Abbreviations

Se: Selenium
SeY: Selenium yeast
Sel-Met: Selenium methionine
PMECs: Porcine mammary epithelial cells
TRX: Thioredoxin reductase
GPX: Glutathione peroxidases
TXNRD: Thioredoxin reductase family
DIOs: Deiodinase family
JNK: Jun-n-kinase
MAPKs: Mitogen-activated protein kinases.
